# Sanitary Privies

**Published:** 1912-02

**Authors:** 


Sanitary Privies. The September 1911 issue of the *Bulletin of the Illinois State Board of Health* is largely devoted to the safe disposal of human excrement. The Lumsden, Roberts and Stiles privy is adopted as the most practical solution of the problem for farms and other places where there is no sewerage system. This consists of an ordinary back house, the vault of which is replaced by a water-tight oil barrel or similar receptacle. Near the top of this, a T pipe is fitted, both ends of the T being covered with wire gauze and the long pipe discharging the excess of liquid into a covered kettle or similar receptacle. Full specifications are provided so that, except for the T pipe and the two receptacles, the construction is within the skill of any amateur carpenter.

*Farmer's Bulletin* No. 459 by L. O. Howard of the U. S. Department of Agriculture is included with the above. This discusses the House Fly. This sort of popular education in sanitation ought to produce results.

